# Xiao Chai Hu Tang for Peptic Ulcers: A Systematic Review and Meta-Analysis of Randomized Controlled Trials

**DOI:** 10.1155/2021/6693677

**Published:** 2021-04-29

**Authors:** Min Li, Wenchao Dan, Hui Zhang, Yong'en Yun, Qingyong He

**Affiliations:** ^1^Department of Cardiology, Guang'anmen Hospital, China Academy of Chinese Medical Sciences, Beijing 100053, China; ^2^Beijing University of Chinese Medicine, Beijing 100029, China; ^3^Henan University of Chinese Medicine, Zhengzhou 450000, China

## Abstract

A peptic ulcer (PU) is a digestive disorder most commonly found in clinical practice. An oriental herbal formula, Xiao Chai Hu Tang (XCHT), has been used to treat PU for an extended period in China. The effectiveness and safety of XCHT in treating peptic ulcers was evaluated using a systematic review of randomized controlled trials (RCTs). Studies were systematically retrieved from CNKI, Embase, Medline, PubMed, SinoMed, VIP, Wanfang, and Web of Science. The following information was extracted from the relevant RCTs: the clinical efficacy rate, recurrence rate, clinical efficacy of traditional Chinese medicine, and the adverse effects. 13 RCTs, including 1334 patients, were included in this review. The meta-analysis showed that treatment with XCHT was superior to conventional pharmacotherapy (CPT) in improving the clinical efficacy rate (RR: 1.20, 95% confidence intervals (CIs): 1.08–1.34, *P*=0.0007), poor appetite (RR: 0.30, 95% CI: 0.15–0.61, *P*=0.0009), abdominal distension (RR: 0.61, 95% CI: 0.39–0.96, *P*=0.03), vomiting (RR: 0.33, 95% CI: 0.19–0.55, *P* < 0.0001), and stomach pain (RR: 0.36, 95% CI: 0.19–0.68, *P*=0.002) and reducing adverse events (RR: 0.23, 95% CI: 0.07–0.69, *P*=0.009). XCHT considerably increased the total clinical efficacy rate (RR: 1.22, 95% CI: 1.15–1.30, *P* < 0.00001) as both monotherapy and adjunctive therapy. The recurrence rate (RR = 0.29; 95% CI: 0.16–0.52, *P* < 0.0001) was remarkably decreased in the XCHT plus CPT group. The meta-analysis did not show a significant beneficial effect of XCHT compared with CPT in reducing the recurrence rate (RR = 0.45; 95% CI: 0.07–3.10, *P*=0.42) and acid reflux (RR: 0.76, 95% CI: 0.47–1.23, *P*=0.26). Our findings show that XCHT can treat peptic ulcers as part of an alternative medicine approach.

## 1. Introduction

PU is defined as ulceration inside the gastrointestinal (GI) mucosa, which occurs due to erosion induced by peptic acid. Conversely, gastric ulcer (GU) and duodenal ulcer (DU) [1] mostly happen in the stomach or proximal duodenum but can occur in the esophagus as well as Meckel's diverticulum [2]. The lifelong prevalence of PU is 5 to 10%, and the incidence is 0.1 to 0.3% per year in the general population [3]. For many years, PU's primary treatment has been triple or quadruple therapy, a western medicine (WM) approach. However, patients treated by WM have a comparatively high recurrence rate after surgery and ongoing treatment effects.

Furthermore, patients can be affected by several adverse reactions during WM treatment [4, 5]. Therefore, there is an urgent need for new treatment options that can alleviate PU. In recent years, strategies focused on traditional Chinese medicine (TCM) have attracted increasing interest.

TCM interventions, including but not limited to Chinese Herbal Medicine (CHM) and treatments such as acupuncture, are commonly applied in Asia and are gradually being accepted worldwide [6]. Randomized controlled trials te articles were screened by two independent investigators (ML and HZ) based on the title and a have shown that herbal medicine can alleviate symptoms, relieve pain, and prevent and treat diseases [7]. However, there is little evidence available regarding the use of CHM to treat PU. Although few RCTs of CHM interventions have been conducted, there is some evidence that Sho Saiko to/Xiao Chai Hu Tang (XCHT) can address many digestive diseases. XCHT is a type of formulary medicine that consists of seven kinds of herbal medicines: Bupleuri Radix, Ginseng Radix, Glycyrrhizae Radix et Rhizome, Pinelliae Tuber, Scutellariae Radix, Zingiberis Rhizoma Recens, and Zizyphi Fructus. XCHT was initially reported as “Treatise on Cold Pathogenic and Miscellaneous Diseases” in a traditional Chinese text written by Zhang Zhongjing in the third century. Existing pharmacologic studies have shown that XCHT can significantly reduce damage to the gastric mucosa of bile acid and hinder gastric acid secretion [8]. Zhou et al. reported that many GU cases had been cured by Modified Xiao Chai Hu Tang (MXCHT). The effective treatment of MXCHT has been widely reported [9]. Several studies have shown that XCHT performs better than standard WM in terms of its therapeutic effectiveness against PU with a lower recurrence rate, higher curative rate, higher *Helicobacter pylori* (Hp) eradication rate, and a significantly reduced ulcer area [10]. Nonetheless, the application of XCHT, which can supplement the limits of standard treatment for PU, has not yet been thoroughly reviewed.

This is the first meta-analysis and systematic review that aims to evaluate the effect of XCHT on clinical efficacy rate, recurrence rate, the clinical efficacy of traditional Chinese medicine, and the adverse effects. Comparison types in RCTs include XCHT alone or XCHT plus WM compared with WM. The patients with PU in the control group are treated with recommended conventional medicine (proton pump inhibitors (PPIs), histamine-2 receptor antagonists (H2RAs), protective agents for gastric mucosa, and drugs targeting *H. pylori*).

## 2. Materials and Methods

This meta-analysis and systematic review follows the Cochrane Handbook criteria [11] and Preferred Reporting Items for Systematic Reviews and Meta-Analyses [12].

### 2.1. Search Strategies

This meta-analysis was registered on PROSPERO (CRD42020209106). A comprehensive electronic search was carried out using four Chinese databases and four English databases. The English databases were Cochrane, Embase, PubMed, and Web of Science. The four Chinese databases were the Wanfang Database, SinoMed, the VIP information database, and the Chinese National Knowledge Infrastructure. The included studies were collected by two team members and all were published before September 2020. The following search terms were used: (“Peptic Ulcer” [MeSH Terms] OR “peptic ulcers” [Title/Abstract] OR “ulcer peptic” [Title/Abstract] OR “ulcers peptic” [Title/Abstract] OR “gastroduodenal ulcer” [Title/Abstract] OR “gastroduodenal ulcers” [Title/Abstract] OR “ulcer gastroduodenal” [Title/Abstract] OR “ulcers gastroduodenal” [Title/Abstract] OR “marginal ulcer” [Title/Abstract] OR “marginal ulcers” [Title/Abstract] OR “ulcer marginal” [Title/Abstract] OR “ulcers marginal” [Title/Abstract] OR “PUD” [Title/Abstract] OR “PU” [Title/Abstract] OR “gastric ulcer” [Title/Abstract] OR “duodenal ulcer” [Title/Abstract]) AND (“xiao chaihu tang” [Title/Abstract] OR “xiao chaihu decoction” [Title/Abstract] OR “XCHT” [Title/Abstract] OR “sho saiko to” [Title/Abstract] OR “sho saiko to” [Title/Abstract] OR “sho” [Title/Abstract]) AND (“randomised controlled trial” [Title/Abstract] OR “clinical trial” [Title/Abstract] OR “clinical study” [Title/Abstract]). In addition, to identify other relevant research, a record of reclaimed article references was hand-searched. All included studies were comprehensively read. Each database was searched individually.

### 2.2. Selection Criteria

All the studies collected by our team members were added to Endnote. After removing duplicate articles, all the candidate articles were screened by two independent investigators (ML and HZ) based on the title and abstract. This review included all RCTs that investigated the outcome of XCHT among patients with PU. Comments, editorials, letters, methodological reports, observational studies, opinion pieces, and traditional literature reviews were excluded.

#### 2.2.1. Type of Subjects

Adult subjects of any gender or ethnicity with PU were included. Two diagnostic criteria for PU were thoroughly applied for this research (first, the American International Health Alliance's Protocol for Diagnosis and Treatment of PU in Adults (2002) and second the Guiding Principle of Clinical Research on New Drugs of TCM, issued by the Ministry of Public Health of China, in 1993 and 2002).

#### 2.2.2. Type of Study

Only RCTs that evaluated the utility and reliability of XCHT to treat PU were eligible to be included. All blinded and non-blinded RCTs of both languages were included. Trials with a sample size of less than ten were excluded as well as any duplicated reports.

#### 2.2.3. Type of Intervention

The patients in the treatment group were mainly treated with XCHT or XCHT combined with WM. The patients were treated with standard WM within the control group. Modified XCHT (MXCHT) prescribed based on syndrome-characterized TCM was acceptable. Practitioners stated that MXCHT merely joined the initial herbs, leading to a similar effect as the original XCHT.

#### 2.2.4. Type of Outcome Measures

The clinical efficacy rate was defined as the primary clinical endpoint. The total clinical efficacy rate of PU treatment would be calculated as (clinical cure + markedly effective + effective)/total number of participants. The efficacy of RCTs was assessed by Criteria for Diagnosis and Curative Effect of TCM Clinical Diseases [13]. First, ulcer healing status would be classified into four categories:

*(1) Clinical Cure*. The ulcer disappeared completely, the local was slightly red, there was no obvious edema, and the conscious symptoms disappeared or basically disappeared.

*(2) Markedly Effective*. The ulcer disappeared or turned into a scar tissue stage, and symptoms such as anorexia, gastric pain, acid reflux, and vomiting disappeared.

*(3) Effective*. The ulcer changed from the active stage to the healing stage, the area of the ulcer was reduced by ≥50%, and symptoms such as anorexia, gastric pain, acid reflux, and vomiting were alleviated.

*(4) Noneffective*. There was no change in the ulcer, or the ulcer area was reduced by <50%. There was no apparent improvement in symptoms such as anorexia, gastric pain, acid reflux, and vomiting.

The secondary results included the number of adverse events, recurrence rate, and clinical effect of TCM symptoms, including vomiting, stomachache, acid reflux, appetite loss, and abdominal distension.

All disagreements on data collection and report choice were explored and resolved through a discussion.

### 2.3. Data Extraction

Relevant data from the RCTs was collected by two investigators separately with a data abstracted criteria sheet and then cross-checked. Each study's information included the baseline equilibrium, blinded experiment, control groups, evaluation indicators, issued year, names of the authors, randomization method, research samples, results, treatment course, type of ulcers, and interventional measures and efficacy follow-up duration. Two investigators (WD and YY) separately rated the included RCTs and collected the data. All disagreements were resolved by a third author (QH).

### 2.4. Quality Assessment of Research

The overall evidence rank was evaluated based on the measurement of the main results according to the methodological analysis in the Grading of Recommendations, Assessment, Development, and Evaluation (GRADE) [14]. The entire evidence rank was categorized using the GRADE evaluation into four grades: high, moderate, low, and exceptionally low. The following could downgrade the class of evidence: fortuity, irrelevance, inaccuracy, risks of bias, and publication bias. As wide confidence intervals (CIs) indicate a lack of accuracy, the RCT's evidence rank could be downgraded by one or two grades. A summary of the main results was conducted with GRADEpro 3.6.

### 2.5. Data Analysis

Data from the included studies was summed to generate quantitative summaries using the Cochrane Collaboration Review Manager (RevMan 5.4). The results were gathered using mean differences with 95% CIs. The chi-squared test and the *I*^2^ statistic (0%–40%: can be secondary; 30%–60%: may represent moderate heterogeneity; 50%–90%: may represent substantial heterogeneity; 75%–100%: considerable heterogeneity) were applied to evaluate the heterogeneity. A fixed-effect model was used when there was no heterogeneity (*P* > 0.1, *I*^2^ < 50%). Otherwise, the random effect model was a more suitable match in general. *P* value <0.05 was considered statistically significant.

### 2.6. Risk of Bias Assessment

The industrial classification of qualified research was assessed separately using the Cochrane Collaboration's tool comprising the following seven domains:

1. Random sequence generation (selection bias)
2. Allocation concealment (selection bias)
3. Blinding of participants and personnel (performance bias)
4. Blinding of the outcome assessment (detection bias)
5. Incomplete outcome data (attrition bias)
6. Selective reporting (reporting bias)
7. Other sources of bias

The following three types of bias risk were used across all domains: unclear risk of bias, low risk of bias, and high risk of bias. Based on the types mentioned above, the quality of each study was classified as follows: fair: low risk for two items; weak: low risk for fewer than two items; good: low risk for more than two items. The final scores were agreed upon by all the authors.

## 3. Results

### 3.1. Research Selection

In total, 653 potentially eligible articles were collected, of which 321 duplicates were removed. From the remaining 332 studies assessed in detail, 186 studies were precluded for one or more of the following reasons.

(a) Case reports and reviews
(b) Summary of clinical classifications
  - Not clinical trials
  - Trials treated with non-drug therapy such as massage, acupuncture, and other non-drug therapy
  - Not relevant to PU

Thus, 133 more studies were precluded by further evaluation due to the following:

- Not RCTs
- No relevant interventions
- No relevant outcome

**Figure 1:**
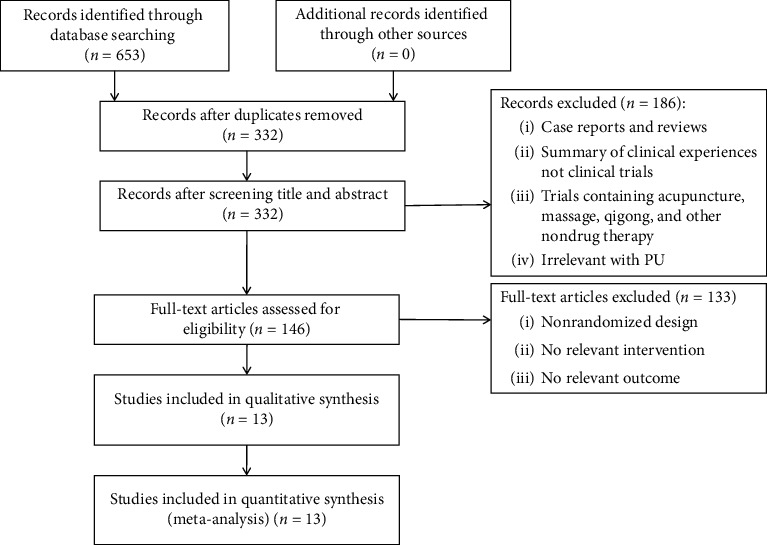
PRISMA flowchart detailing the data identification, screening, eligibility, and inclusion.

Eventually, 13 studies [15–27] were identified. The research selection process and reasons for excluding articles are shown in Figure 1.

### 3.2. Features of the Included Studies

Table 1 outlines the features of the selected studies. A total of 1334 patients aged 30–59 years were included, of whom 669 and 665 were in the intervention and control groups, respectively. There were 13 studies with two arms published from 2009 to 2017. The sample sizes ranged from 30 to 100, with a trial duration ranging from 28 to 75 days. The participants of the intervention group in 5 studies [15, 17, 22, 23, 26] were prescribed XCHT, and 8 studies [16, 18–21, 24, 25, 27] were XCHT plus conventional pharmacological therapy (CPT). The control group of 13 studies received conventional treatment with WM. Among these, 5 studies (638 participants) [15, 17, 22, 23, 26] compared XCHT with CPT. The other 8 studies (*n* = 696 participants) [16, 18–21, 24, 25, 27] compared XCHT plus CPT with the same Western medications used alone. 13 studies mentioned the clinical efficacy rate, and 4 studies [14, 15, 18, 20] reported the adverse effects. 5 studies [17, 21, 24, 26, 27] reported the recurrence rate, and 4 studies [15, 22, 25, 26] reported the clinical efficacy of TCM symptoms. The consecutive duration from 1 to 12 months was reported in 7 studies [15, 17, 18, 21, 24, 26, 27].

### 3.3. Risk of Bias Assessment

**Figure 2:**
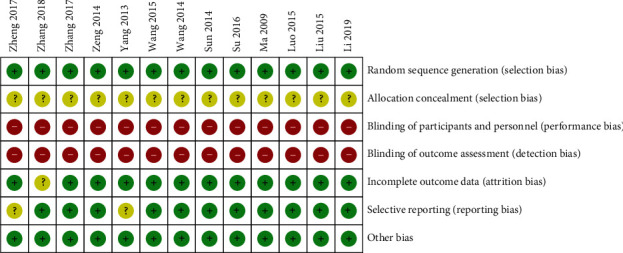
Risk of bias summary.

**Figure 3:**
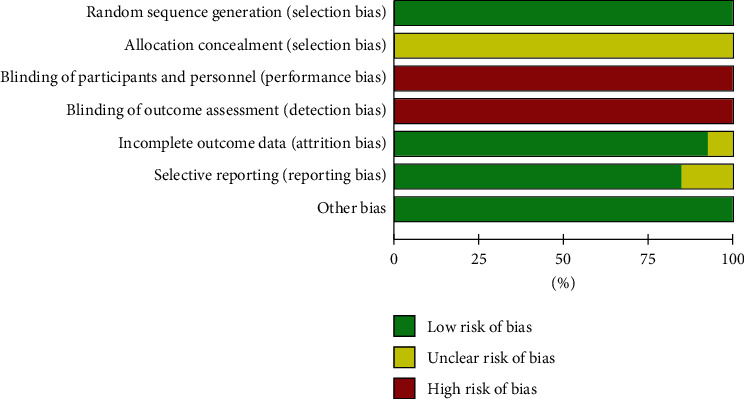
Risk of bias graph.

The Cochrane Collaboration assessment tool was applied to evaluate the risk of bias of the included studies. The evaluation outcomes were listed in Figures 2 and 3. The risk of bias summary of RevMan 5.4 presented the results of the risk of bias assessment. Although all studies discussed the randomization method used, only 5 trials [15, 16, 20, 22, 25] used a random sequence generation approach, including electronic random number tables. There was no blinding design and allocation concealment in the selected studies. No research protocols were included in the RCTs. 11 studies [15–18, 20–22, 24–27] with selective reporting were evaluated as low risk. The 2 remaining studies [19, 23] were assessed as having unclear results due to only including a vague description. All 13 studies were counted as having a low risk of bias because there was no clear evidence showing other sources of bias. Information on the patients that dropped out of the studies was not provided.

### 3.4. Quality Assessment Using GRADE

Tables 2 and 3 showed the results of the quality assessment using GRADE. The evidence supporting the differences between XCHT plus CPT and CPT for clinical efficacy rate, adverse events, and the recurrence rate was high and moderate. Comparing XCHT with CPT, differences in the clinical efficacy rate were apparent. Furthermore, the recurrence rate was too high, and adverse events were moderate, while evidence for vomiting, stomachache, acid reflux, appetite loss, and abdominal distension was low.

### 3.5. Statistical Analysis

A sensitivity analysis of the evaluation indicators (clinical efficacy rate, recurrence rate, and adverse events) that were heterogeneous among the studies was conducted after excluding studies one by one to verify the stability of the analysis results. The sensitivity analysis results of the clinical efficacy rate, recurrence rate, and adverse events did not detect significant differences, indicating that the analysis results were stable.

### 3.6. Clinical Efficacy Rate

**Figure 4:**
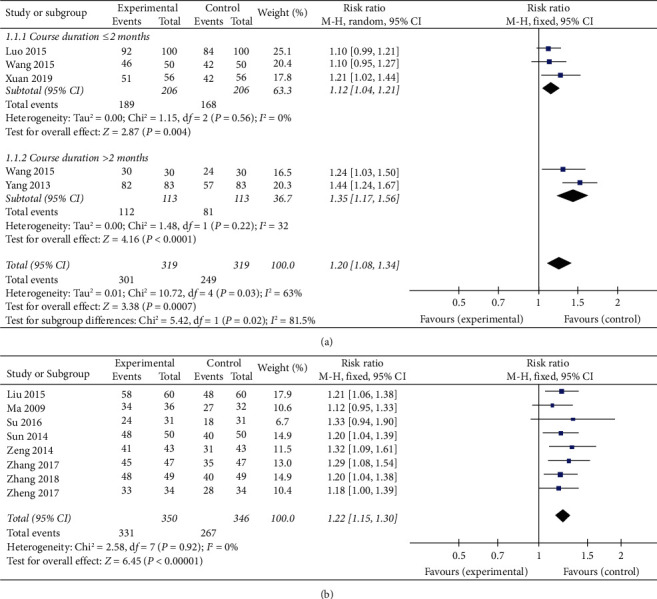
Meta-analysis of the clinical efficacy rate of (a) XCHT and (b) XCHT + CPT.

13 studies reported the clinical efficacy rate. The clinical efficacy rate was compared between those receiving XCHT and routine treatment in 5 studies [15, 17, 22, 23, 27]. Five studies showed heterogeneity (*I*^2^ = 63%). Thus, a random-effects model was applied. There was significant improvement compared to XCHT with CPT (RR: 1.20, 95% CI: 1.08–1.34, *P*=0.0007). No significant changes occurred in the sensitivity analyses. Nevertheless, the outcome states an essential difference (Figure 4(a)) in the subgroup concerning the course duration.

The clinical efficacy rate was compared between those receiving XCHT plus CPT and CPT alone in 8 studies [16, 18–21, 24, 25, 27]. The meta-analysis demonstrated that XCHT plus routine treatment was significantly better at enhancing the clinical efficacy rate than regular treatment (RR: 1.22, 95% CI: 1.15–1.30, *P* < 0.00001, *I*^2^ = 0%) (Figure 4(b)).

### 3.7. Recurrence Rate

**Figure 5:**
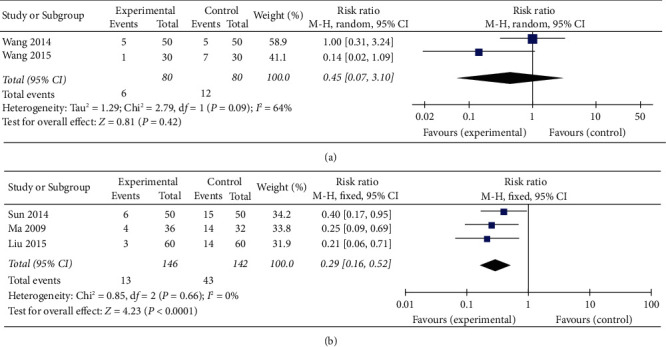
Meta-analysis of the recurrence rate of (a) XCHT and (b) XCHT + CPT.

Comparing the recurrence rate between patients who received XCHT treatment and CPT was conducted on 160 subjects in 2 RCTs [17, 26]. Marked heterogeneity was observed among the studies (*χ*^2^ = 2.79, df = 1, *P*=0.09, *I*^2^ = 64%), thus requiring the application of a random-effects model. In contrast to the CPT group (RR = 0.45; 95% CI: 0.07–3.10, *P*=0.42) (Figure 5(a)), the meta-analysis did not show any essential favourable effect in the XCHT group. The heterogeneity was more than 50%, but subgroup and sensitivity analyses could not be carried out using only two RCTs.

Three studies [21, 24, 27], with a total of 288 subjects, compared XCHT plus CPT with CPT alone, using the recurrence rate as the outcome measure.  Figure 5 shows the combined effect located on the left side of the forest plot. A pooled analysis showed that XCHT plus CPT resulted in a reduced recurrence rate compared with CPT alone (RR = 0.29; 95% CI: 0.16–0.52, *P* < 0.0001, *I*^2^ = 0%; Figure 5(b)).

### 3.8. Clinical Efficacy of TCM Symptoms

**Figure 6:**
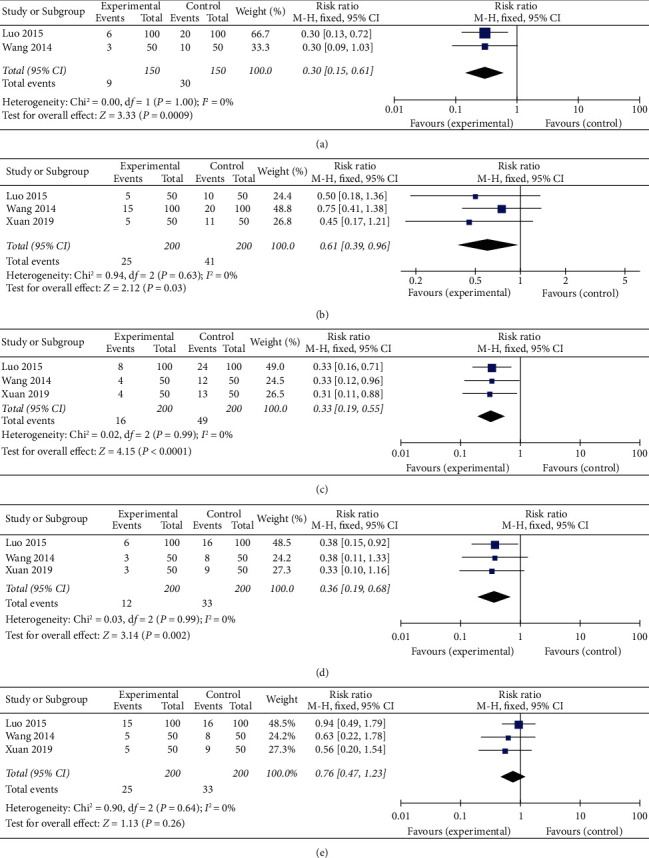
Meta-analysis on the clinical efficacy of TCM symptoms. (a–e) show the comparisons and meta-analysis results on the clinical efficacy of TCM symptoms between the XCHT and CPT groups. (a) Poor appetite. (b) Distention. (c) Vomit. (d) Stomach pain. (e) Acid reflux.

3 trials [15, 22, 26] that provided data on the clinical effect of TCM symptoms (such as poor appetite, acid reflux, and vomiting) were included in the meta-analysis. XCHT treatment was reported to be better than CPT in terms of poor appetite (RR: 0.30, 95% CI: 0.15–0.61, *P*=0.0009, *I*^2^ = 0%) in 2 trials [22, 26] with 300 patients (Figure 6(a)). 3 studies of 400 patients with abdominal distension showed that XCHT had a therapeutic effect (RR: 0.61, 95% CI: 0.39–0.96, *P*=0.03, *I*^2^ = 0%; Figure 6(b)). Vomiting relieved significantly with the XCHT arm in 3 trials (RR: 0.33, 95% CI: 0.19–0.55, *P*=0.0001, *I*^2^ = 0%; Figure 6(c)). The meta-analysis of these 3 trials revealed that XCHT significantly relieved stomach pain compared to the controls (RR: 0.36, 95% CI: 0.19–0.68, *P*=0.002, *I*^2^ = 0%; Figure 6(d)). Among the 3 trials that recorded acid reflux, there was no significant difference between the XCHT and CPT groups, as shown in the meta-analysis (RR: 0.76, 95% CI: 0.47–1.23, *P*=0.26, *I*^2^ = 0%; Figure 6(e)).

### 3.9. Adverse Events

**Figure 7:**
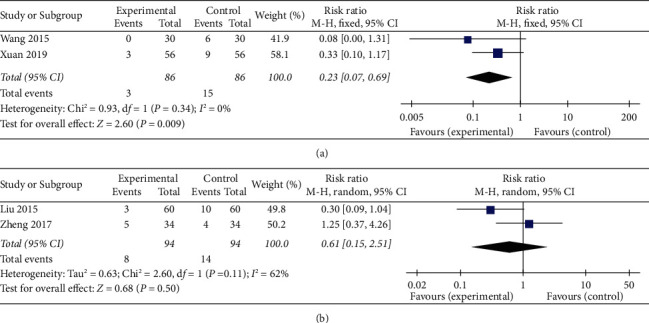
Meta-analysis on adverse events of (a) XCHT and (b) XCHT + CPT.

Among the 13 studies, 4 studies [15, 17, 19, 21] reported the adverse events that occurred along with the treatment, in which a total count of 11/669 (1.64%) and 29/665 (4.36%) patients suffered an adverse event in the trial and control groups, respectively. No adverse effects were observed in the other nine studies. 5 cases were reported in 1 trial [19]. These were gastrointestinal reactions, including nausea, dry mouth, vomiting, and diarrhea in the XCHT plus CPT group, with the side effects disappearing after the drug was discontinued. Nervous system issues, such as dizziness, were the second most common symptom, with 3 cases in the XCHT group and 9 cases in the CPT group in another trial [25]. The adverse events in all studies were mild in both the control and XCHT groups. According to a meta-analysis of 2 studies [15, 17], XCHT was better accepted than CPT because of its lower side effects (RR: 0.23, 95% CI: 0.07–0.69, *P*=0.009, *I*^2^ = 0%) (Figure 7(a)).

However, the other 2 trial results [19, 21] were not homogeneous (*χ*^2^ = 2.60, df = 1, *P*=0.11, *I*^2^ = 62%). Hence, the source of heterogeneity by subgroup and the study model's stability could not be determined based on only 2 studies. The meta-analysis showed no significant difference between the XCHT plus CPT and CPT groups (RR: 0.61, 95% CI: 0.15–2.51, *P*=0.50) (Figure 7(b)).

### 3.10. Publication Bias

Based on the Cochrane guidelines, publication bias was not fulfilled as the number of RCTs for meta-analysis purposes of every major result measure was no more than nine.

## 4. Discussion

PU is related to diet, stress, and a hypersecretory acid environment. However, the epidemiology of PU is changing due to alcohol and smoking abuse, Hp contamination, and widespread application of non-steroidal anti-inflammatory drugs (NSAIDs). A small number of patients with Hp inflammation or those on aspirin or NSAIDs develop PU, suggesting that the specific sensitivity of drug toxicity and bacterial harm are significant to start mucosal damage [28, 29]. Patients with GU typically present with nausea, vomiting, weight loss, and postprandial abdominal pain. Patients who have DU often present with abdominal pain and a feeling of hunger. PU is characterized by self-healing, recurrence, and a comparatively high incidence rate of up to 50% in short-term cases [30]. Gastric mucosal injury, NSAIDs, Hp infection, and excessive gastric acid secretion are associated with increased PU risk. PU can reduce patients' quality of life and increase health care costs.

WM has been predominately used to treat GU, but the drugs available are limited in their efficacy. For instance, proton pump inhibitors are considered efficient drugs for PU treatment [31]. However, they have many adverse effects, such as headaches, rashes, and gastrointestinal disturbances. Among the selected RCTs, the following symptoms were occasionally reported: angina, muscle weakness, mental disorders, hypersensitivity reactions, severe allergic reactions, kidney damage, liver failure, and reversible confused states [32]. Moreover, drug resistance of Hp has increased due to the widespread use of antibiotics [33].

### 4.1. Summary of Evidence

13 RCTs of 1334 patients with PU, including GU and DU, were collected for analysis. Our study's main finding is that XCHT therapy is superior to CPT, with a better clinical efficacy rate and fewer side effects. Similarly, XCHT therapy was excellent to CPT except in terms of acid reflux. XCHT could reduce PU symptoms in patients much more effectively. As an alternative medicine, XCHT appeared to be associated with an improved clinical efficacy rate and fewer adverse events. The included studies showed that XCHT combined with CPT seemed to be more effective at reducing the recurrence rate than CPT alone. Additionally, XCHT is safe to apply and is generally well-tolerated by patients. Therefore, XCHT could successfully treat patients with PU, improve the healing of the ulcer, and reduce the recurrence rate.

### 4.2. Limitations

To the best of our knowledge, the present systematic review and meta-analysis is the first to assess the effects of XCHT on PU and provide a thorough synthesis of results from RCTs. However, the present study has some limitations. First, every trial selected was performed in Asia, which limits diversity and inclusion. Further research with multi-center RCTs of XCHT for PU is needed to expand the research worldwide. Second, some of the included RCTs were of low methodological quality. None of the RCTs detailed the proper distribution occultation clearly, which can cause selection bias.

Moreover, a lack of double-blinding of participants and personnel can result in detection and performance biases. However, it can be challenging to the blind in XCHT RCTs, as the smell, taste, and color of XCHT are apparent. A placebo with no medical reaction can imitate XCHT, which is not beneficial for improving the rationality or benefit of medical proof and lowering selective reporting bias. Third, the clinical efficacy rate, including clinical cure, markedly effective, effective, and noneffective, is generally considered result measurements. However, based on the clinical symptoms, the clinical efficacy rate assessment may be vague and subjective, limiting its usefulness. Finally, latent clinical heterogeneity can be caused by different XCHT medications being used and different doses.

### 4.3. Suggestion of Practices

XCHT treatment for patients with PU appears to be safe and efficacious. The essential pathogenic factors of PU include both excess and deficiency syndrome based on TCM theory. The actual pathological factors mainly include (i) Qi stagnation, (ii) cold coagulation, (iii) food accumulation, (iv) amp heat, and (v) blood stasis. The main pathological factors of deficiency are (i) Qi (yang) deficiency and (ii) Yin deficiency [34]. Qi stagnation resulting from unstable emotions was the most crucial factor that could injure the liver [35].

Moreover, PU is located in the stomach, closely related to the liver and spleen [36]. Nevertheless, the long-term disease course can cause blood stasis and lead to blood deficiency [37]. Saikosaponin-a (Ssa) and saikosaponin-d (Ssd), which are related to Gan Qi regulation, were revealed in a recently published study [38]. According to Matsuta et al., XCHT can protect the gastric mucosa, which can be related to the activation of defense factors and suppression of attack factors [39]. XCHT should be considered as a candidate for clinical trials and herbal prescriptions.

### 4.4. Suggestions for Future Research

Sufficient patients should be included in future clinical trials with blinded, statistical, and proper randomization methods applied. Based on the limitations mentioned above, the latest international guidelines and a consistent, standard diagnosis method should be used to select patients in future RCTs.

The inclusion and exclusion criteria should be explicitly stated. Age groups should be clearly defined. A description of the outcome and baseline data for the control and treatment groups must be displayed. The adoption and application of efficacy rate scales should be in agreement with the latest updated global guidelines for future statistical investigation. Patients should undergo long-term follow-up to observe any potential adverse reactions. Medical research needs to be registered in advance and provide the trial date at the end of the experiment.

Modern medical research on PU treated with XCHT has progressed considerably. In modern pharmacology, it is well acknowledged that GU is caused by attack and defense factors of unbalanced mucosa [40]. Liu et al. stated that XCHT exerted protective effects on GU models. The mechanisms were anti-secretory, reduced the secretion of pro-inflammatory mediators, elevated the levels of anti-inflammatory cytokines, increased epidermal growth factor (EGF) activation, and upregulated the presentation of heat shock protein 70 kD (HSP-70), p-serine-threonine kinase (p-AKT), and proliferating cell nuclear antigen (PCNA) [17]. Yoshio found that XCHT suppressed ethanol-induced gastric lesions [41]. According to a study, B. Radix, a significant component of XCHT, had an anti-ulcer action and suppressed gastric acid secretion [42].

## 5. Conclusion

This meta-analysis and systematic review supports the use of XCHT for PU as part of an alternative medicine approach to a certain extent. However, due to clinical heterogeneity, the results of this review should be treated cautiously. Furthermore, RCTs of specific XCHT, supervised with quality control for PU patients, are progressing.

## Figures and Tables

**Table 1:** Characteristics of the 13 included RCTs.

ID	Sample size (T/C)	Mean age (years) (T/C)	Diagnostic standards	Type of ulcer (D/G/C) (T/C)	Intervention	Comparison	Course duration (days)	Outcome	Adverse effects
Li et al.	112	T: 42.62 ± 6.71	The diagnostic methods and criteria for peptic ulcer disease (2010)	T: 30/26/0	XCHT	Routine treatment	60	Clinical efficacy rate, the clinical efficacy of TCM symptoms	T: 3
	(56/56)	C: 42.59 ± 6.56		C: 32/24/0					C: 9

Wang	60	T: 42.8 ± 3.7	Guidance principle of clinical study on new drug of traditional Chinese medicine (1993) and criteria of diagnosis therapeutic effect of diseases and syndromes in traditional Chinese medicine (1994)	T: 11/19/0	XCHT	Routine treatment	75	Clinical efficacy rate, recurrence rate	T: 0
	(30/30)	C: 42.5 ± 3.4		C: 10/20/0					C: 6
Luo	200	T: 56.2 ± 3.4	The practice of internal medicine (unclear) and guidance principle of clinical study on new drug of traditional Chinese medicine (1993)	NR	XCHT	Routine treatment	28	Clinical efficacy rate, the clinical efficacy of TCM symptoms	NR
	(100/100)	C: 53 ± 6.4							

Yang et al.	166	NR	Internal medicine teaching (unclear) and guidance principle of clinical study on new drug of traditional Chinese medicine (1993)	NR	XCHT	Routine treatment	75	Clinical efficacy rate	NR
	(83/83)								

Wang	100	T: 42.24 ± 7.31	Internal medicine (unclear)	T: 33/17/0	XCHT	Routine treatment	56	Clinical efficacy rate, recurrence rate, the clinical efficacy of TCM symptoms	NR
	(50/50)	C: 41.86 ± 6.93		C: 18/32/0					

Zhang	98	T: 34.13 ± 2.13	NR	NR	XCHT + routine treatment	Routine treatment	28	Clinical efficacy rate	NR
	(49/49)	C: 34.78 ± 2.12		NR					

Zeng	86	T: 32 ± 2.5	NR	NR	XCHT + routine treatment	Routine treatment	30	Clinical efficacy rate	NR
	(43/43)	C: 33 ± 2.3		NR					

Zheng et al.	68	T: 37.52 ± 3.25	Practice of internal medicine (unclear)	NR	XCHT + routine treatment	Routine treatment	28	Clinical efficacy rate	T: 5
	(43/43)	C: 37.05 ± 3.12		NR					C: 4

Zhang	94	T: 55.35 ± 7.24	Guidance principle of clinical study on new drug of traditional Chinese medicine (2002) and practice of internal medicine (2010)	T: 19/25/3	XCHT + routine treatment	Routine treatment	28	Clinical efficacy rate	NR
	(47/47)	C: 53.13 ± 6.68		C: 25/15/7					

Liu	120	T: 36.5 ± 3.9	Guidance principle of clinical study on new drug of traditional Chinese medicine (2002) and internal medicine (unclear)	T: 19/30/11	XCHT + routine treatment	Routine treatment	75	Clinical efficacy rate, recurrence rate	T: 3
	(60/60)	C: 44.5 ± 2.6		C: 21/29/10					C: 10

Sun	100	T: 32 ± 2.5	Guidance principle of clinical study on new drug of traditional Chinese medicine (1993) and practice of internal medicine (unclear)	T: 18/22/10	XCHT + routine treatment	Routine treatment	30	Clinical efficacy rate, recurrence rate	NR
	(50/50)	C: 33 ± 2.4		C: 19/23/8					

Su et al.	62	T: 56.2 ± 5.4	Guidance principle of clinical study on new drug of traditional Chinese medicine (2002) and practice of internal medicine (2002)	T: 9/20/2	XCHT + routine treatment	Routine treatment	28	Clinical efficacy rate, the clinical efficacy of TCM symptoms	NR
	(31/31)	C: 53.4 ± 6.2		C: 9/20/2					

Ma	68	T: 35.2	Guidance principle of clinical study on new drug of traditional Chinese medicine (2002) and practice of internal medicine (1997)	T: 12/20/6	XCHT + routine treatment	Routine treatment	28	Clinical efficacy rate, recurrence rate	NR
	(36/32)	C: 37.3		C: 11/17/4					

T, treatment group; C, control group; XCHT, Xiao Chai Hu Tang; NR, not reported; D, duodenal ulcer; G, gastric ulcer; C, compound ulcer.

**Table 2:** Assessment of the study quality using GRADE (XCHT plus CPT compared to CPT).

Outcomes	Illustrative comparative risks^*∗*^ (95% CI)		Relative effect (95% CI)	No. of participants (studies)	Quality of the evidence (GRADE)
	The assumed risk with the comparator	The corresponding risk with intervention			
Clinical efficacy rate	772 per 1000	941 per 1000 (887 to 1000)	RR 1.22 (1.15 to 1.3)	696 (8 studies)	⊕⊕⊕⊕ high
Adverse events	149 per 1000	91 per 1000 (22 to 374)	RR 0.61 (0.15 to 2.51)	188 (2 studies)	⊕⊕⊕⊕ high
Recurrence rate	303 per 1000	88 per 1000 (48 to 157)	RR 0.29 (0.16 to 0.52)	288 (3 studies)	⊕⊕⊕⊝ moderate

^∗^The control risk is based on the median risk of the control group of each study. The intervention risk (and its 95% CI) is based on the control risk in the control group and the relative effect of the intervention (and its 95% CI).CI: confidence interval, RR: risk ratio. GRADE Working Group grades of evidence: (1) High certainty: we are very confident that the true effect lies close to that of the estimate of the effect; (2) Moderate certainty: we are moderately confident in the effect estimate. The true effect is likely to be close to the estimate of the effect, but there is a possibility that it is substantially different; (3) Low certainty: our confidence in the effect estimate is limited. The true effect may be substantially different from the estimate of the effect; (4) Very low certainty: we have very little confidence in the effect estimate ^⊕⊕⊕⊕^represents high-quality evidence; ^⊕⊕⊕⊖^represents moderate-quality evidence.

**Table 3:** Assessment of the study quality using GRADE (XCHT compared to CPT).

Outcomes	Illustrative comparative risks^*∗*^ (95% CI)		Relative effect (95% CI)	No of participants (studies)	Quality of the evidence (GRADE)
	The assumed risk with the comparator	The corresponding risk with intervention			
Total effective rate	781 per 1000	937 per 1000 (843 to 1000)	RR 1.2 (1.08 to 1.34)	638 (5 studies)	⊕⊕⊕⊕ high
Adverse events	150 per 1000	68 per 1000 (11 to 465)	RR 0.45 (0.07 to 3.1)	160 (2 studies)	⊕⊕⊕⊝ moderate
Recurrence rate	174 per 1000	40 per 1000 (12 to 120)	RR 0.23 (0.07 to 0.69)	172 (2 studies)	⊕⊕⊕⊕ high
Vomiting	245 per 1000	81 per 1000 (47 to 135)	RR 0.33 (0.19 to 0.55)	400 (3 studies)	⊕⊕⊝⊝ low
Stomachache	165 per 1000	59 per 1000 (31 to 112)	RR 0.36 (0.19 to 0.68)	400 (3 studies)	⊕⊕⊝⊝ low
Acid reflux	165 per 1000	125 per 1000 (78 to 203)	RR 0.76 (0.47 to 1.23)	400 (3 studies)	⊕⊕⊝⊝ low
Abdominal distension	205 per 1000	125 per 1000 (80 to 197)	RR 0.61 (0.39 to 0.96)	400 (3 studies)	⊕⊕⊝⊝ low
Appetite loss	200 per 1000	60 per 1000 (30 to 122)	RR 0.3 (0.15 to 0.61)	300 (2 studies)	⊕⊕⊝⊝ low

^∗^The control risk is based on the median risk of the control group of each study. The intervention risk (and its 95% CI) is based on the control risk in the control group and the relative effect of the intervention (and its 95% CI).CI: confidence interval, RR: risk ratio. GRADE Working Group grades of evidence: (1) High certainty: we are very confident that the true effect lies close to that of the estimate of the effect; (2) Moderate certainty: we are moderately confident in the effect estimate. The true effect is likely to be close to the estimate of the effect, but there is a possibility that it is substantially different; (3) Low certainty: our confidence in the effect estimate is limited. The true effect may be substantially different from the estimate of the effect; (4) Very low certainty: we have very little confidence in the effect estimate. ^⊕⊕⊕⊕^ represents high-quality evidence; ^⊕⊕⊕⊝^ represents moderate-quality evidence; ^⊕⊕⊖⊖^ represents low-quality evidence.

## Data Availability

Any data that were analyzed and extracted or supporting information is included in this manuscript. Detailed datasets or any other information are available on reasonable request from the corresponding author.
